# Enhanced Electrochemical Performance of Supercapacitors via Atomic Layer Deposition of ZnO on the Activated Carbon Electrode Material

**DOI:** 10.3390/molecules26144188

**Published:** 2021-07-09

**Authors:** Chongrui Wu, Fuming Zhang, Xiangshang Xiao, Junyan Chen, Junqi Sun, Dayakar Gandla, Yair Ein-Eli, Daniel Q. Tan

**Affiliations:** 1 Department of Materials Science and Engineering, Guangdong Technion Israel Institute of Technology, 241 Daxue Road, Jinping District, Shantou 515063, China; chongrui.wu@gtiit.edu.cn (C.W.); fuming.zhang@gtiit.edu.cn (F.Z.); xiao.xiangshang@gtiit.edu.cn (X.X.); chen.junyan@gtiit.edu.cn (J.C.); sun.junqi@gtiit.edu.cn (J.S.); dayakar.gandla@gtiit.edu.cn (D.G.); 2 Department of Materials Science and Engineering and Grad Technion Energy Program (GTEP), Technion-Israel Institute of Technology, Haifa 3200003, Israel

**Keywords:** atomic layer deposition, porous activated carbon, zinc oxide, high voltage, supercapacitors

## Abstract

Fabricating electrical double-layer capacitors (EDLCs) with high energy density for various applications has been of great interest in recent years. However, activated carbon (AC) electrodes are restricted to a lower operating voltage because they suffer from instability above a threshold potential window. Thus, they are limited in their energy storage. The deposition of inorganic compounds’ atomic layer deposition (ALD) aiming to enhance cycling performance of supercapacitors and battery electrodes can be applied to the AC electrode materials. Here, we report on the investigation of zinc oxide (ZnO) coating strategy in terms of different pulse times of precursors, ALD cycles, and deposition temperatures to ensure high electrical conductivity and capacitance retention without blocking the micropores of the AC electrode. Crystalline ZnO phase with its optimal forming condition is obtained preferably using a longer precursor pulse time. Supercapacitors comprising AC electrodes coated with 20 cycles of ALD ZnO at 70 °C and operated in TEABF_4_/acetonitrile organic electrolyte show a specific capacitance of 23.13 F g^−1^ at 5 mA cm^−2^ and enhanced capacitance retention at 3.2 V, which well exceeds the normal working voltage of a commercial EDLC product (2.7 V). This work delivers an additional feasible approach of using ZnO ALD modification of AC materials, enhancing and promoting stable EDLC cells under high working voltages.

## 1. Introduction

As the conventional and traditional energy resources are depleting, there is an increasing need to develop renewable, low-cost, and efficient power and energy sources. To satisfy the needs of modern society, supercapacitors with fast charging/discharging rates, long cycle life, and high-power density attract worldwide research and commercial attention. An electrical double-layer capacitor (EDLC) is a common device showing great potential in fast energy discharge applications. However, the low operating voltages, high leakage current, and low energy density of supercapacitors limit their applications [1,2]. The degradation at the interface of electrodes and electrolytes is of a concern when raising the operation voltages [3,4]. To prolong and extend the cycling life of the EDLC and battery electrodes, researchers are directing their efforts at surface modification of the electrodes as a most effective way to reach the target.

Recently, the atomic layer deposition (ALD) thin-film technique has gained significant attention. It allows the deposition of a desired conformal coatings at an atomic scale on various complex substrates [5,6]. Two or more chemical vapor precursors react in the ALD chamber, forming an ultrathin inorganic layer on the substrates to be modified. The operating temperature for ALD can be relatively low; this is an advantage, enabling the deposition of the desired film on substances and materials that are temperature sensitive [7,8]. It is a suitable technique for coating activated carbon (AC) electrodes, possessing limited ion accessibility in organic electrolyte [9]. Our group has developed various oxide coatings by ALD for activated carbon (AC) electrodes, enhancing the cycling stability of supercapacitors, while maintaining a low impedance at high voltage windows [10]. Hong et al. deposited an ultrathin Al_2_O_3_ using ALD on a commercial AC electrode, widening the operating voltage window of EDLC cells to 3.5 V in organic electrolytes [11]. More research on metal oxide deposition using ALD also sheds some light on improving supercapacitor electrodes [12,13,14,15,16]. In addition, Cho et al. deposited Al_2_O_3_ and TiO_2_ thin films on the surface of spinel-phase oxide (5 V Li-Mn-Ni oxide spinel, LMNO) using ALD, resulting in remarkably different electrochemical performance than non-coated LNMO nanowire electrode [17]. Ein-Eli and co-workers have applied different metal-fluorides (MgF_2_, AlF_3_, and LiF) ALD coating on LMNO powder material and reported a massive enhancement in cycling stability [18,19,20]. These results demonstrated that the ALD technique is the most effective tool to cope with the severe cycling deterioration of high operational voltage materials in lithium-ion batteries and supercapacitors. However, most importantly, each ALD design and coating must be well-tailored to protect the substrate [21]. In this sense, commercial supercapacitors made of YP−50 activated carbon electrodes often suffer from electrolyte decomposition at the solid-liquid interface, leading to lower capacity retention at an operating voltage of >3V. It is a rational choice to tackle these challenges via an ALD metal oxide coating method to prolong the cycling life of the AC-based EDLCs enabling a higher operational voltage window.

Zinc oxide (ZnO) is a typical semiconducting material, which may accelerate ion transportation while acting as a protective layer on the electrodes compared to non-conductive aluminum oxide coating. Its semiconductor nature could be advantageous over the dielectric oxide in terms of low impedance and fast discharge, and possibly an additional capacitance may be harvested. The ZnO coating effect on electrochemical performance for different energy storage materials has been reported earlier [22,23,24]. Wang et al. discovered that zinc oxide and titanium dioxide on lithium-rich layered cathodes result in more stable cycling performance and thermal stability than the pristine samples. [23]. Zhu et al. coated Li_2_MnSiO_4_/C electrode with ZnO layer by a wet chemistry method. Compared to bare electrodes, ZnO-coated Li_2_MnSiO_4_/C shows better performance. [25]. Zhao et al. deposited ultrathin ZnO, ZrO_2_, and Al_2_O_3_ coatings (~1 nm) on the surfaces of LiMn_2_O_4_, reporting the highest final capacity when the electrodes were coated with 6 ZnO ALD cycles [26]. It was concluded that ALD parameters play a vital role in ZnO formation and subsequently on the recorded electrochemical properties [27,28].

Thin ZnO coating was applied on other non-carbon planar substrates extensively; such materials are sapphire, quartz, silicon, glass, borosilicate, and reviewed by Tynell and Karppinen [24]. However, ALD of ZnO has not shown much success on activated carbon electrodes for EDLC application. Here, in the current work, we explore the ALD of ZnO processed on AC electrodes and determine the experimental conditions enabling the enhancement of commercial YP−50 AC substrate. Therefore, we report here on an effective and most appropriate ALD process scheme enabling the fabrication of ZnO-coated AC nanostructures. The positive effect of ZnO ALD is pronounced by significantly improving the cycling retention of the EDLC cells when operated up to a voltage of 3.2 V.

## 2. Materials and Methods

### 2.1. Electrode and Electrolyte Materials

AC powders used in this work were YP−50F (Kuraray), and PVDF binders and carbon black were purchased from Sigma Aldrich. AC electrodes were fabricated on Al foils using the doctor blade casting technique with an automatic thin-film coater (MTI corporation, Hefei, China). A 35-μm thick cellulose NKK TF4035 of 75% porosity (Nippon Kodoshi, Kochi, Japan) was used as a separator. The electrolyte salt TEABF_4_ (Sigma Aldrich) was placed in a glove box filled with high purity argon (<0.01 ppm O_2_ and <0.01 ppm H_2_O), then dissolved in the Acetonitrile (AN) in a concentration of 1 mol/L.

### 2.2. ALD ZnO Coating Process

ALD oxide coating was carried out using a Benchtop GESTar XT-ALD system (Arradiance, Inc). Zin oxide coating was obtained at various deposition temperatures using Diethyl Zinc (DEZ) purchased from Nanjing Ai Mou Yuan Scientific Equipment Co. Lt. and high-performance liquid chromatography grade H_2_O precursors following the chemical reaction described below:

Zn(CH_2_ CH_3_)_2_ + H_2_O → ZnO + 2CH_3_ CH_3._

The typical growth rate for the film is 0.5–1.5 Å per cycle for zinc oxide material. The reaction sequence within each deposition cycle was: (i) exposure of the substrate to DEZ; (ii) purge to remove the un-reacted precursors and the byproducts; (iii) exposure of the substrate to H_2_O; (iv) purge to remove the un-reacted precursors and the byproducts detailed by Song et al. [10,29].

**Figure 1:**
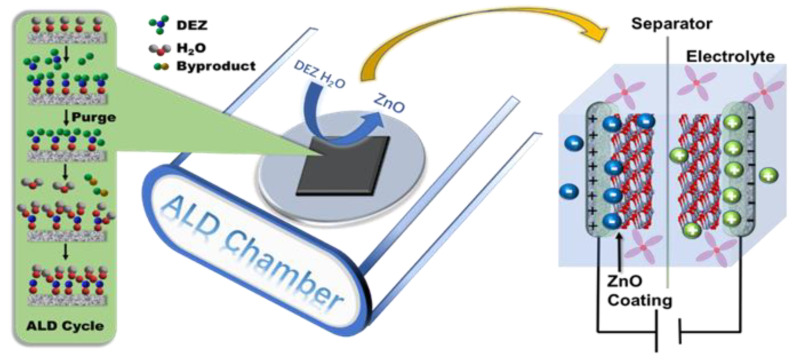
Schematic illustration of ALD coating and EDLC cell modified with crystalline ZnO.

The AC electrodes were fixed onto a silicon wafer with Kapton tapes (Figure 1). Each ALD process was conducted in the chamber set at 70, 120, and 150 °C separately and a constant argon flow rate of 10 sccm was applied. The pulse time was 400 ms, and purge time was 10 s in each deposition cycle. Detailed parameters of the different ALD depositions are shown in Table 1. Those coated samples were transferred to clean polyethylene zip bags for a storage after coating.

### 2.3. Microstructural Analysis

The crystallographic structures of bare AC and ALD oxides-coated AC electrodes were examined using a Rigaku Smartlab 9 X-ray diffractometer at a scan rate of 6°/min using 150 mA current, 40 kV voltage, and copper target. The surface morphology and particle size were characterized using a ZEISS Sigma−500 field emission scanning electron microscopy (FESEM). The elemental distribution and mapping were obtained using Energy Dispersive Spectroscopy (EDS, BRUKE XFlash−6130, Berlin, Germany). Transmission electron microscopy (TEM) images were captured on a JEM2100 instrument at an acceleration voltage of 200 kV to analyze the deposition features on the carbon surface. Quantachrome Autosorb-iQ2-MP (Quantachrome Instruments, Florida, USA) and nitrogen isotherms were used to test specific surface areas by BET tests with degassing at 250 °C for 3 h for each 36 mg sample. The DFT (density functional theory) and HK (Horvath-Kawazoe) methods from N2 adsorption-desorption isotherms (at 77.4 K) were employed to verify the surface area and porosity. The elemental distribution and functional changes were explored using X-ray photoelectron spectroscopy (Thermo Scientific ESCALAB 250Xi).

### 2.4. Electrochemical Measurements

YP−50F AC powder (~85%), super P carbon black (~10%), and polyvinylidene difluoride (~5%) in N-methyl-2-pyrrolidinone solution were mixed for 10 min in a vacuum mixer for a homogeneous slurry. The slurry was loaded onto Al foil and spread using the doctor blade technique to form a film. This film was vacuum dried at 120 °C for 12 h and then pressed at 10 MPa to form a dense electrode sheet. Afterwards, the electrode sheet was cut into disks of 1.12 cm^2^ using a disk cutter (MSK-T10).

The electrochemical performance of the AC and ALD ZnO-coated AC electrodes were evaluated based on a symmetrical supercapacitor device with CR2032 coins. The cyclic voltammetry and Galvanostatic charge-discharge (GCD) measurements between 2 to 50 mA cm^−2^ using the Gamry electrochemical workstation (Interface 1010E, USA). EIS data were recorded with 5 mV amplitude potential within a 10 kHz−0.01 Hz frequency range.

## 3. Results and Discussion

### 3.1. Deposition of ZnO ALD on AC Electrodes

The deposition of ZnO via the ALD technique may end up at amorphous and crystalline structures depending on the processing conditions. Previous reports emphasized the importance of ALD deposition temperatures when using various substrates, and reported on the temperature dependence of ZnO phase formation, O/Zn ratio, and resistivity [24]. Our anticipation is to obtain the crystalline ZnO phase for lower electrical resistivity and complete coverage in the AC electrodes via suitable ALD schemes, as shown in Figure 1. 

**Figure 2:**
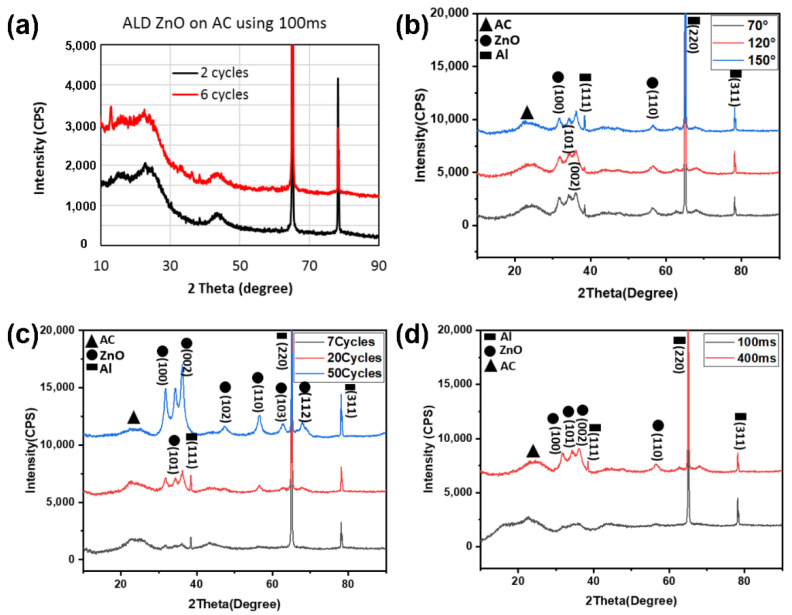
XRD patterns of ALD ZnO-coated AC under different conditions: (**a**) 100 ms at 120 °C for 2 and 6 cycles; (**b**) 100 ms and 400 ms at 70 °C for 20 cycles; **(c**) 400 ms at 150 °C for 7, 20, and 50 cycles; (**d**) pulse time of 400 ms for 20 cycles, at 70, 120, and 150 °C.

We first attempted to obtain a deposition of a ZnO thin layer on a tortuous AC substrate at various temperatures. Figure 2a depicts XRD results of ALD ZnO deposition at 120 °C using 100 ms pulse time for DEZ. With an increase in the ALD cycles from 2 to 6, one can see in the 2θ spectrum no ZnO peak formation, except the carbon and aluminum substrate peaks. Increasing the number of ALD cycles to 20 results in a broad XRD signal between 30 and 40 degrees, indicating ZnO’s appearance, as shown in Figure 2b. Yet, the deposited material does not grow into the crystalline ZnO phase, using the same pulsing time (100 ms). 

Following that, we increased the pulse time of the DEZ precursor to 400 ms. We observed the formation of clear crystalline peaks for the same number of ALD cycles and deposition temperature. These peaks between 2θ angle of 30 and 40° are labeled as (100), (002), (101) and marked inside the dashed green square (Figure 2b). The appearance of the crystalline phase at the lower deposition temperature is attributed to the extended pulse time for ZnO ALD coating on AC electrodes. When using 400 ms, it turns out that even at 7-ALD cycles at the same deposition temperature (150 °C), the ZnO phase is already apparent, as shown in Figure 2c. Increasing the cycle numbers to 20 and 50 under these conditions further sharpen the ZnO peaks, indicating a complete crystallization of the ZnO phase. These crystalline peaks do not change much when decreasing the deposition temperature from 150 to 70 °C. Therefore, a crystalline ZnO phase can be formed on AC electrodes using 400 ms for several ALD cycles at temperatures as low as 70 °C.

**Figure 3:**
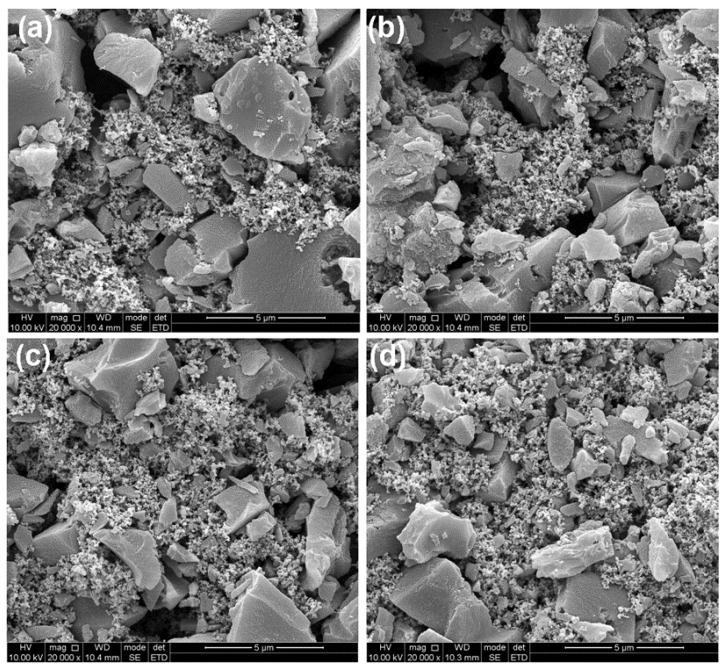
SEM images of Bare AC and ZnO-coated AC at 70 °C: (**a**) Bare AC; (**b**) 2-cycle ZnO; (**c**) 6-cycle ZnO; (**d**) 20-cycle ZnO.

SEM images presented in Figure 3 illustrate the coated AC particle morphology after fabricating on an aluminum foil electrode. There are bulk structures and small particles between them in each image, probably the carbon black and PVDF binder. A similar morphology in all samples being deposited with 20-cycle ZnO was obtained, and no observable alteration in the AC electrode microstructure was detected. 

**Figure 4:**
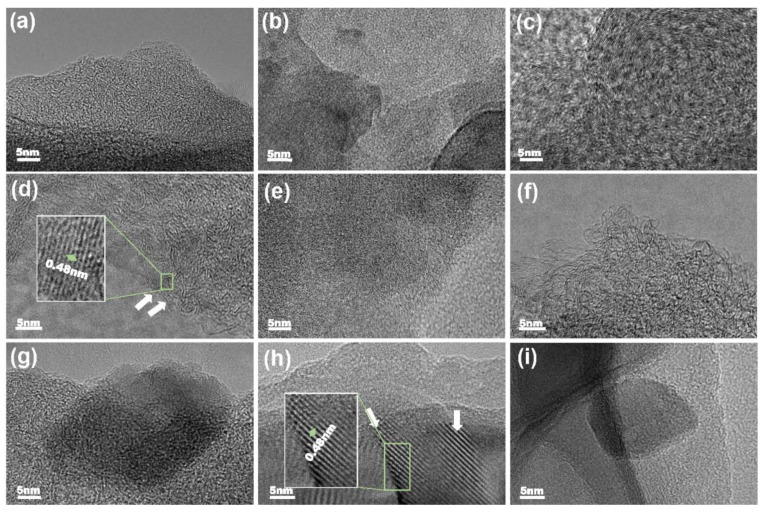
TEM images of Bare AC (**a**–**c**), 2-cycle ZnO-coated AC at 70 °C (**d**–**f**), and 20-cycle ZnO-coated AC at 70 °C (**g**–**i**).

To confirm the formation and existence of ZnO on the AC substrates, we analyzed these samples using HRTEM and EDS. The detailed characterization is shown in Figure 4, illustrating the uneven and rough AC surface. Bare AC is shown in Figure 4a–c, and 2-cycle ZnO samples in Figure 4d–f do not present much difference. For 20-cycle ZnO, dark-colored fringes on the edges of the AC particles are observable at different locations (as marked with arrows), indicating most probably the presence of ZnO coating on the AC surface.

**Figure 5:**
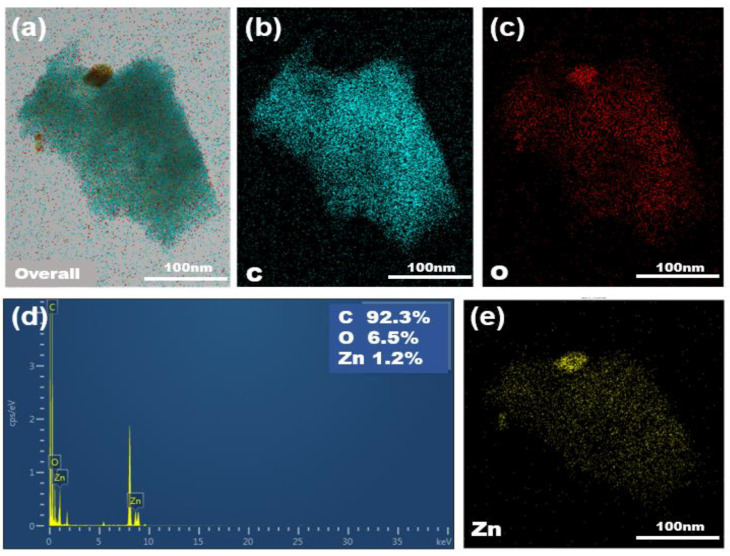
EDS images of Figure 3g: (**a**) Overall, (**b**) Carbon distribution, (**c**) Oxygen distribution, (**d**) EDS mapping, (**e**) Zinc distribution.

More evidence of ZnO coating achieved at 150 °C is provided with the use of EDS analysis, as shown in Figure 5d,e. Zn and O elements are shown everywhere in the elemental mapping, and a ZnO particle is seen as well on the top of the AC sample. These images confirm the effectiveness of the ALD ZnO deposition.

**Figure 6:**
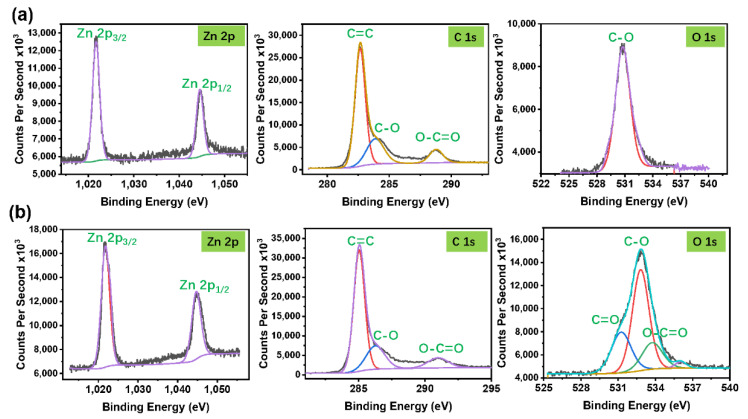
Zn 2p, C_1s_, and O_1s_ core level XPS spectra of (**a**) 2-cycle and (**b**)20-cycle ZnO-coated AC at 100 ms and 70 °C.

XPS technique was used to reveal the surface elemental composition and oxygen content on the AC electrodes coated with 2-cycle ZnO and 20-cycle ZnO at 70 °C. Figure 6a,b show the typical Zn peaks (Zn2p1/2 and Zn2p3/2), direct evidence of the presence of ZnO even at the early stage of phase formation as indicated in the XRD pattern (Figure 2a). Additional coating cycles do not show the change in Zn duplet peaks; however, more carbon-oxygen bonds are generated, indicating increased oxygen content. The relevant peaks can be de-convoluted into three peaks of C=O (531.5 eV), C-O (532.6 eV), and O-C=O (533.7 eV), respectively. At 2-cycle coating, C-O is the dominant bond, and the other two are not detected. However, in the 20-cycle coating, the peak becomes much wider, and other peaks can be de-convoluted, evidence that oxygen is extensively involved in ZnO crystalline phase.

As is shown in Figure 6, more ALD cycles lead to the introduction of C=O and O-C=O bonds. Accordingly, the AC electrode coated with 20-cycle ZnO gives rise to higher capacitance and longer charge-discharge time than AC coated with 2-cycles ZnO. This difference may be associated with the active sites and better ionic mobility provided by oxygen functional groups.

### 3.2. Electrochemical Performance of ALD ZnO-Coated AC Electrodes

#### 3.2.1. Dependence on ALD Coating Cycle

**Figure 7:**
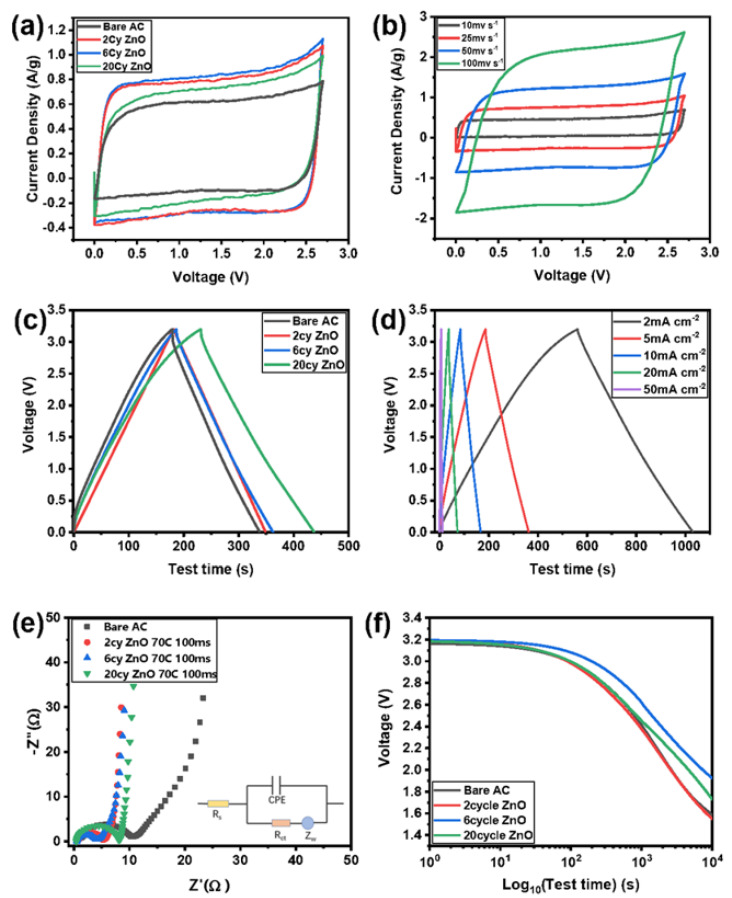
Electrochemical characterization of ZnO-coated AC at 100 ms and 70 °C: (**a**) at different ALD coating cycles at the scan rate of 25 mV/s; (**b**) CV loop of 6-cycle ZnO coating at different scan rates; (**c**) GCD curves at different ALD coating cycles; (**d**) Charge-discharge profiles of 6-cycle ZnO coating at 3.2 V using different current densities; (**e**) EIS spectrum of various ZnO ALD coated AC; (f) Self-discharge behavior of various ZnO ALD coated AC.

The electrochemical performance of bare AC and ZnO-coated AC electrodes was first initiated with those using the pulse time of 100 ms. The symmetric coin cells are fabricated using TEABF_4_/acetonitrile electrolyte for various tests. Figure 7a depicts the cyclic voltammetry of the electrodes with different coating thicknesses. Up to 2.7 V, all samples show a square-like shape without much distortion. Like the 2-cycle coated material, 6-cycle ZnO-coated AC delivers the best performance, albeit both give rise to a higher capacitance than bare AC. Further evaluation of the 20 cycles ZnO present an opposite trend: the capacitance now turns to a lower value. This result shows that a suitable thin coating contributes to the capacitance in EDLC more significantly. Too thick ZnO coating using the pulse time of 100 ms is not favorable in terms of the C-V loop area. Figure 7b shows the CV curves of 6-cycle ZnO coating at various scan rates. The loss of capacitance due to the high scan rate is insignificant (Figure 7b). In Figure 7c, 20-cycle ZnO-coated samples have a longer charge-discharge time when charging to 3.2 V, indicating its higher capacitance compared with the other three materials. The GCD curves of 6-cycle ZnO coating at various current densities exhibit the typical triangular shape with high linearity and symmetry, illustrating the excellent charge and discharge reversibility of ALD ZnO-coated samples Figure 7d. 

In Figure 7c, 20-cycle ZnO-coated samples have a longer charge-discharge time when charging to 3.2 V, meaning its higher capacitance than the other three materials. Figure 7e presents electrochemical impedance spectra (EIS) data comparison between the ZnO-coated and pristine uncoated samples. It is easy to relate it to the result that the coated samples have lower impedance, and the trend is identical to the performance in the C-V behavior. The self-discharge behavior is also studied for all the samples, as shown in Figure 7f. An open-circuit voltage was used for 12 h after charging at a constant voltage of 3.2 V for 1 h. As observed from the curves, the voltage of 6-cycle coated samples decreases slowly due to time. The 6-cycle coated samples still maintain 2V after 10,000 s, followed by the 20-cycle coated sample.

**Figure 8:**
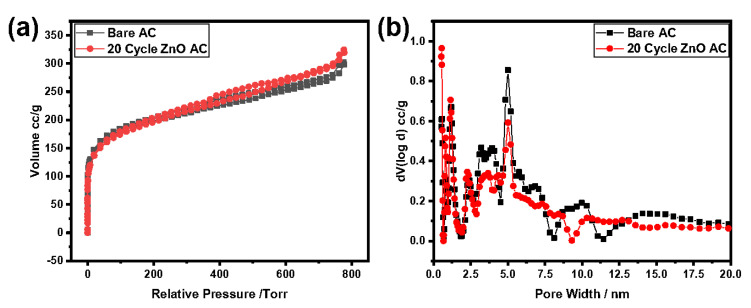
(**a**) Nitrogen adsorption and desorption isotherms; (**b**) pore size distribution for bare AC and 20-cycle ZnO at 100 ms and 70 °C.

The increased capacitance by applying ZnO coating is attributed to the maintenance of the high surface area of AC. ALD coating of fewer than 20 cycles is still thin enough in comparison with the micropore structure. The DFT and HK methods from N_2_ adsorption-desorption isotherms reveal the surface area and porosity of pristine and ZnO-coated AC electrodes. Figure 8 shows a minor loop for both the bare and ZnO-coated AC samples indicating the existence of mesopores and negligent difference in terms of pore size distribution. In other words, conformal ZnO coatings at 70 °C and 100 ms are realized and do not block the micropores on the electrode surfaces. 

#### 3.2.2. Dependence on ALD Pulsing Time and Temperatures

**Figure 9:**
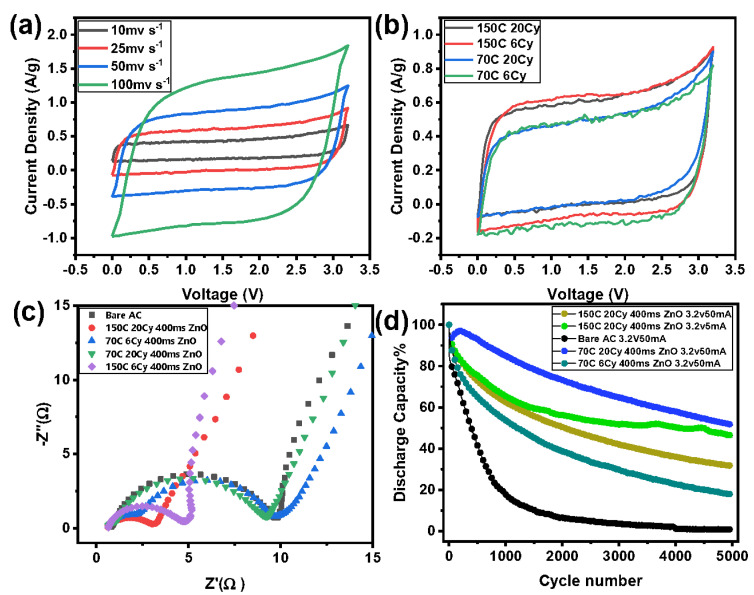
Comparison between bare AC and ZnO-coated AC samples using 400 ms pulse time: (**a**) C-V of 20-cycle ZnO-coated at 150 °C; (**b**) C-V of ZnO-coated sample at 70 °C and 150 °C; (**c**) Electrochemical impedance spectrum of bare AC and coated AC at various conditions; (**d**) Cyclic stability at 3.2 V and /50 mA for 5000 cycles of various samples.

We studied the relationship between the electrochemical performance and ZnO phase formation on AC using a longer pulse time (400 ms). Figure 9a reveals the 3V C-V loop of AC electrodes coated with 20-cycle ZnO using 400 ms at 150 °C. The ALD cycle number does not show different results (Figure 9b). However, coating at 150 °C results in a higher capacitance than those at 70 °C. The long pulsing time also benefits their impedance behavior. At 400 ms pulse time, all the ZnO-coated AC electrodes, irrespectively of the number of ALD cycles and ALD temperature, showed lower charge transfer resistance (R_ct_) values than the pristine bare AC (9.1 Ω). The R_ct_ values of the 6- and 20-cycle ALD coated electrodes at 150 °C were found to be 4.3 Ω and 2.3 Ω, respectively, whereas at 70 °C, the R_ct_ values were 4.3 Ω and 2.3 Ω, respectively. The Lower R_ct_ at 150 °C in comparison to 70 °C can be attributed to the minimal differences in the extent of crystalline ZnO phase formation, affecting the ion migration rate at the electrode and electrolyte interface. Such behavior is also observed in ZnO nanoparticle-decorated bacterial nanowires. [21,22].

ALD ZnO coating also benefits the cycling retention of the AC-based supercapacitors operating at higher voltages. This characteristic is desirable since a device using TEABF_4_ electrolyte cannot be used at 3.2 V, due to the severe electrolyte degradation initiated at the AC-electrolyte interface. Figure 9d summarizes the cyclic life of the bare AC and AC samples coated at different conditions. When cycling at 3.2 V and 50 mA, the bare AC electrodes perform poorly, failing quickly after 1000 cycles. On the other hand, the AC electrodes coated with ZnO exhibit a significant improvement in retention at similar testing conditions. In particular, 20-cycle ALD ZnO at 70 to 150 °C for 400 ms performs better in cycling retention. Yet, the cyclic capacitance stability remains to be further improved from 60 to 90%.

### 3.3. ALD of ZnO on AC Powders Compared to ALD of ZnO on Electrodes 

**Figure 10:**
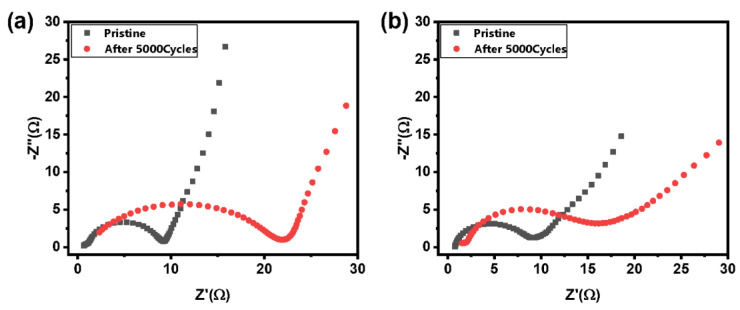
Impedance behavior of AC-based EDLC cells with different ZnO coating schemes (**a**) 20-cycle ZnO ALD coated on AC electrodes; (**b**) 20-cycle ZnO ALD coated on AC powders.

The reduction in impedance by applying ALD of ZnO at 20 cycles for 400 ms at 150 °C is now being applied and tested to ALD of ZnO on AC powder before electrode fabrication. We aimed to understand better the impact of the ALD processing sequence on the overall impedance of the cells. The impedance of the coated AC powder-based EDLC is also apparently reduced compared with the ALD of the composite AC electrodes (Figure 10). After 5000 charge-discharge cycles at 3.2 V, the cells comprising AC powders coated with ALD of ZnO only show a minor increase in the impedance: from 9 to 15 Ω; whereas the cells containing ALD coated composite AC electrodes exhibit a significant increase in the impedance. It can be attributed to homogeneous and complete (and possibly compact) ZnO coverage on the AC powder. 

Overall, the lower impedance values for ZnO-coated AC electrodes compared with the pristine uncoated AC (Table 2) implies the enhanced electron/ion diffusion rate across the interface. The ZnO coating cycles or thickness should be minimal when depositing at a lower temperature (70 °C) for a shorter pulse time (100 ms) since 20-cycle coating causes a higher impedance than 2-cycle coating. One may understand this phenomenon based on the intrinsic n-type conductivity originated from Zn interstitials and impurities in the ZnO crystal. Increasing the ALD deposition temperature up to 220°C generally decreases the resistivity and the stoichiometry of the ZnO film. Thus, it also results in lower resistivity (~10^−3^ Ω cm) than the pristine AC materials [12,13]. 

## 4. Conclusions

This work reports on the successful deposition of crystalline ZnO thin layer on an activated carbon substrate (electrode and powder) by controlling the pulse time of precursors and deposition temperature within the range of 70 and 150 °C. The Zn-based precursor’s pulse time is the most critical parameter and should be longer than 100 ms. Longer pulse time (for example, 400 ms) readily results in the formation of a crystalline ZnO and a lower impedance in EDLC cells. An ultrathin ZnO coating (6–20 cycles) is identified by XRD patterns, indicating that the crystalline phase enhances the electrochemical performance. The novel EDLC cells assembled with ZnO ALD coated AC electrodes demonstrated the enhanced cyclic capacitance retention (50%) under a higher operation voltage window (3.2 V) and the impedance reduction (3–10 Ohm). ZnO coating on powders before fabricating electrodes may improve the AC particle contact, leading to a lower impedance. Although the deposition of ZnO on AC has achieved significant improvements in electrochemical performance (such as impedance, electrochemical window, and capacity), it still requires further improvement in cycle life for future commercialization.

## Figures and Tables

**Table 1:** Activated carbon electrodes subjected to various ALD ZnO coating schemes.

ALD Temperature	70 °C	120 °C	150 °C
DEZ pulse time	100 and 400 ms	100 ms	400 ms
ALD cycle	20	2 and 6	7, 20 and 50

**Table 2:** Impedance comparison among various AC electrode materials.

Impedance of EDLC Cells	Pristine AC	AC-Electrode-Coated with ZnO	AC-Powder-Coated with ZnO
ALD at 70 °C, 20 cycle, and 400 ms	10–20 Ω	<10 Ω	<8 Ω
After 5000 cycles	50 Ω	22 Ω	17 Ω

## Data Availability

The data presented in this study are available in the article.

## Sample Availability

Samples of the ALD ZnO-coated AC are available from the authors.
